# Neighborhood Street Scale Elements, Sedentary Time and Cardiometabolic Risk Factors in Inactive Ethnic Minority Women

**DOI:** 10.1371/journal.pone.0051081

**Published:** 2012-12-07

**Authors:** Rebecca E. Lee, Scherezade K. Mama, Heather J. Adamus-Leach

**Affiliations:** Texas Obesity Research Center, Department of Health and Human Performance, University of Houston, Houston, Texas, United States of America; University of Sao Paulo, Brazil

## Abstract

**Background:**

Cardiometabolic risk factors such as obesity, excess percent body fat, high blood pressure, elevated resting heart rate and sedentary behavior have increased in recent decades due to changes in the environment and lifestyle. Neighborhood micro-environmental, street scale elements may contribute to health above and beyond individual characteristics of residents.

**Purpose:**

To investigate the relationship between neighborhood street scale elements and cardiometabolic risk factors among inactive ethnic minority women.

**Method:**

Women (N = 410) completed measures of BMI, percent body fat, blood pressure, resting heart rate, sedentary behavior and demographics. Trained field assessors completed the Pedestrian Environment Data Scan in participants’ neighborhoods. Data were collected from 2006–2008. Multiple regression models were conducted in 2011 to estimate the effect of environmental factors on cardiometabolic risk factors.

**Results:**

Adjusted regression models found an inverse association between sidewalk buffers and blood pressure, between traffic control devices and resting heart rate, and a positive association between presence of pedestrian crossing aids and BMI (ps<.05). Neighborhood attractiveness and safety for walking and cycling were related to more time spent in a motor vehicle (ps<.05).

**Conclusions:**

Findings suggest complex relationships among micro-environmental, street scale elements that may confer important cardiometabolic benefits and risks for residents. Living in the most attractive and safe neighborhoods for physical activity may be associated with longer times spent sitting in the car.

## Introduction

Ethnic minority women have a higher prevalence of obesity (51.0% of African American and 43.4% of Hispanic versus 33.1% of white women) and hypertension (51.5% of African American and 65.3% of Hispanic versus 47.4% of white women) compared to white women, contributing to disease risk [1] and rising health care costs, which are 30% higher for overweight or obese individuals compared to their normal weight peers. [2] Minority women also are less physically active than white women, putting them at greater risk for chronic health conditions related to physical inactivity [3].

Increases in cardiometabolic risk factors such as obesity, excess percent body fat, high blood pressure, elevated resting heart rate and sedentary behavior may reflect changes in lifestyle. [4], [5] Resettlement from urban, highly walkable settings with easy access to work, and goods and services to suburban neighborhoods that are obesogenic [6] has been blamed for these lifestyle changes and subsequent increases in cardiometabolic risk factors. A study by Frank and colleagues found that participants who preferred car dependent environments were twice as likely to be obese than participants who preferred, and lived in, walkable environments. [7] Another study by Gordon-Larsen and colleagues found that active commuting was positively associated with fitness and inversely associated with BMI, obesity, triglyceride levels, blood pressure, and insulin levels [8].

The understanding of the neighborhood micro-environment and its association with cardiometabolic risk factors is an underrepresented domain in this area of research. Ecologic models of health suggest that micro-environmental factors are more proximal, and hypothesized to be more influential on behavior compared to factors that are more distal. [9] Although suburban type neighborhoods have been associated with increases in cardiometabolic risk factors and decreases in physical activity, particular neighborhood micro-environment factors may still be present in ‘non-walkable’ neighborhoods that can contribute to positive health behaviors. For example, the presence and condition of neighborhood streets and sidewalks can likely have a large impact on health by influencing decisions to walk, or bike for leisure, particularly on the weekend. As well, more pleasant neighborhood surroundings might reduce the perception of daily stressors contributing to improved dietary habits, [10] in turn leading to lower rates of obesity, reduced fat on the body and improved blood pressure and resting heart rates. [11], [12] However, few studies have investigated the relationship between micro-environmental, street scale elements and cardiometabolic risk factors, particularly in the most vulnerable population of minority women.

Sedentary behavior, particularly the amount of time spent sitting, has recently received attention as an independent risk factor for cardiovascular disease (CVD) and mortality. A study by Katzmarzyk and colleagues found a higher risk of all-cause and CVD related mortality across higher levels of sitting time, independent of leisure time physical activity. [13] In another study, time riding in a car and the combination of time spent riding in a car and time spent sitting while watching TV was positively associated with CVD mortality. [14] However, few studies have examined the influence of neighborhood or environment on sedentary behaviors, and of those that have, findings are mixed. A study by Kozo and colleagues found that TV viewing time and driving time were higher in neighborhoods of low walkability. [15] In contrast, Van Dyck and colleagues found that living in a high-walkable neighborhood was associated with more self-reported sitting time and more accelerometer-measured sedentary time. [16] A more recent study by the same group found that land use, walking and biking facilities, traffic safety and few cul-de-sacs contributed to less motorized time, but associations with sitting time were unclear. [17] These mixed and uncertain associations of neighborhood walkability with sedentary behavior suggest that sedentary time is a unique and important cardiometabolic risk factor that warrants investigation of micro-environment street scale elements and their impact on sedentary behavior, particularly associated with sitting while driving and general sitting time on weekdays and weekends.

The purpose of this study was to investigate the relationship between neighborhood street scale elements and cardiometabolic risk factors including BMI, percent body fat, blood pressure, resting heart rate and sedentary behavior. It was expected that neighborhood street scale elements which support physical activity such as safety and attractiveness, and the presence of crossing aids and traffic control devices would be inversely associated with BMI, percent body fat, blood pressure, resting heart rate and time spent sitting on the weekend, but positively associated with time spent in a motor vehicle. These neighborhood elements may have a positive influence on cardiometabolic risk factors even in neighborhoods that are not considered supportive for physical activity at a more macro-level (e.g., high population density, mixed land use). Environmental-level and individual-level data were used to determine the relationship between the environment and cardiometabolic risk factors in African American and Hispanic women.

## Methods

### Participants

Data for this study were collected in 2006–2008 and represent measurement of four hundred ten community dwelling, African American (n = 258) and Hispanic or Latina (n = 152) women who participated in Health Is Power, a multi-site, longitudinal, community based, randomized controlled trial to increase physical activity [18] and to investigate the relationship between neighborhood factors and physical activity adoption and maintenance in Houston and Austin, Texas. Participants who were recruited self-identified as African American or Hispanic or Latina, and were between the ages of 25 and 60 years old, able to read, speak, and write in English or Spanish, not pregnant or planning to become pregnant within the next 12 months, a Harris or Travis County resident, not planning on moving in the next 12 months, physically inactive or doing fewer than 30 minutes of physical activity per day on 3 or more days per week, and free from health conditions that would be aggravated by physical activity. [18] All HIP study assessments, measures and procedures were approved by the Committee for the Protection of Human Subjects at the University of Houston. Data for this study were collected from 2006–2008, and analyses for this study were conducted in 2011.

### Measures

#### Sociodemographics

Items assessing ethnicity, household income, and education were adapted from the Maternal Infant Health Assessment (MIHA) survey, [19] derived from the CDC’s Pregnancy Risk Assessment Monitoring System (PRAMS) Questionnaire. [20] Items have been used with samples representing diverse ethnicities and income levels [21].

#### Cardiometabolic risk factors

Anthropometric measures of body mass index (BMI) and body fat were collected by trained personnel using established protocols. [18], [22] Individual height was measured using a standard stadiometer apparatus with participants’ shoes removed. Body weight, BMI and percent body fat were measured together in both pounds and kilograms using a Tanita TBF-310 body composition analyzer (Tanita, Arlington Heights, Illinois; 2007). Measures were collected twice, with the average of the two measurements used in analyses.

Systolic and diastolic blood pressures were measured using manual aneroid sphygmomanometry by a trained research staff member using established protocols. [23] Participants were asked to sit quietly during measurement with their left arm bared and supported at heart level and their feet flat on the floor. Two readings were taken separated by two minutes and averaged for use in analyses. If the first two readings differed by more than 5 mmHg, a third reading was obtained and averaged.

Resting heart rate was assessed by a trained research team member. Participants were asked to first sit quietly for approximately two minutes. The assessor measured their radial pulse at the left wrist by placing her or his index and middle finger over the underside of the participant’s wrist, below the base of the thumb. Assessors counted beats for one full minute and repeated the procedure for accuracy. An average of the two measurements in beats per minute was used in analyses.

#### Sedentary behavior

Sedentary behavior was measured using items from the International Physical Activity Questionnaire (IPAQ) long form. IPAQ correlations are 0.8 for reliability and 0.3 for validity, indicating its acceptability for measuring physical activity in adults 18 to 65 years of age. [24] The International Physical Activity Questionnaire (IPAQ) long form is typically used to measure work-related, transportation, domestic and leisure-time physical activity. In addition, the instrument measures time spent sitting over the last seven days broken down by time spent sitting in a motor vehicle and time spent sitting during the week and weekend. [25] Sedentary behavior was reported in terms of minutes per day for motor vehicle sedentary time and total minutes during the week and weekend.

#### Environmental measures

The Pedestrian Environmental Data Scan (PEDS) instrument measures street segment environmental features and pedestrian facilities related to walking and cycling. It contains 40 questions that measure macro environment features, such as land use, segment type, and connectivity, [26] and micro-environment features, such as lighting, amenities, and articulation. For this investigation, variables measured pedestrian crossing aids (e.g., pedestrian signals, traffic islands or over/under passes), sidewalk traffic buffers (e.g., fence, trees, grass), traffic control devices (e.g., traffic signal, stop sign or speed bumps), number of path connections, number of travel lanes, posted speed limit, and amenities (e.g., benches, trash cans, street vendors), along with four variables rating the safety and attractiveness of the walking and cycling environment [26].

### Procedures

#### Individual assessments

As previously described, women who met inclusion criteria gave informed consent and completed a baseline (T1) health assessment, where they completed an interviewer administered questionnaire, anthropometric measures of body mass index (BMI = kg/m^2^) and percent body fat, and measures of blood pressure and resting heart rate [18].

### Neighborhood Assessments

Neighborhoods were mapped using Geographical Information Systems (GIS) technology and were defined as the area within an 800 meter radius Euclidean buffer surrounding each participant’s house. Some of the original 410 cases were eliminated from the sample due to poor geocoded matches, as some participants gave misinformation or incomplete address information on baseline survey information or refused to provide home residence address, yielding a final sample of 383 neighborhoods. A random sample of 25% of residential street segments and all arterial street segments within a 400 meter radius were assessed using the PEDS instrument. [27] A street segment is a portion of a street that is intersected by two cross streets, or by a cross street at one end and a dead end at the other. [28] All assessments were conducted by trained research team members in teams of at least two people following established data collection and safety protocols [29], [30], [31], [32].

### Statistical Analyses

All statistical analyses were conducted in SPSS Version 18.0 (SPSS 18.0 for Windows, SPSS Inc, Chicago, Ill) in 2011. Bivariate associations (correlations, cross tabs, t-tests and analyses of variance) were conducted for PEDS variables to determine multicollinearity and reduce the number of potential variables to include in analyses. The process used to select the variables has been previously described, [22] and was based on multicollinearity with other PEDS variables, consistency with previously reported relationships, and extensive field experience of investigators. The final variable selection for this study was guided by a review of current literature to generate hypothesized relationship of environmental variables to cardiometabolic factors and included pedestrian crossing aids, sidewalk traffic buffers, traffic control devices, path connections, travel lanes, speed limit, amenities and safety and attractiveness of the walking and cycling environment.

Street segment data were aggregated to the neighborhood level by taking the mean across street segments for each neighborhood, using established protocols for ecologic analyses. [22], [29], [30], [33] Multiple regression models were used to estimate the effect of aggregated environmental factors on cardiometabolic risk factors including BMI, percent body fat, blood pressure, resting heart rate, and sedentary behavior, controlling for age, education, income, site, and ethnicity. Simultaneous or forced entry was used over hierarchical or stepwise or backward entry to test theory-based, predetermined models rather than sequentially testing variables within the model and allowing models that do not contribute significantly to be removed. [34] Significance for all analyses was set at p<.05.

## Results

### Descriptive Characteristics

Most participants (M = 45.2 years, SD = 9.3) were overweight or obese (M BMI = 34.7 kg/m^2^, SD = 8.5; M BF% = 42.8, SD = 7.1). Roughly half (44.2%) had graduated from college, and nearly half (48.6%) reported an income 401% or greater above the Federal Poverty Level. Women were similar in age by ethnicity and site.

Systolic (t = 2.533, p = .012) and diastolic (t = 2.333, p = .020) blood pressure and resting heart rate (t = −2.197, p = .029) varied by ethnicity. African American women had higher blood pressure, Systolic/Diastolic M = 126.3 mmHg (SD = 19.9)/M = 79.6 mmHg (SD = 9.6) versus M = 122.7 mmHg (SD = 13.3)/M = 77.3 mmHg (SD = 9.6), but lower resting heart rate (M = 72.9 BPM (SD = 8.6) versus M = 74.9 BPM (SD = 9.1) than Hispanic or Latina women. There were no other significant differences in cardiometabolic factors by ethnicity. Women reported spending an average of 97.4 (SD = 110.0) minutes sitting in a car each week, an average of 415.4 (SD = 252.4) minutes sitting during the week, and an average of 323.0, (SD = 228.3) minutes sitting during the weekend.

### Bivariable Associations

PEDS variables were significantly correlated with cardiometabolic factors. As neighborhood sidewalk buffers (r = −.116, p = .026), traffic control devices (r = −.118, p = .023), and amenities (r = −.111, p = .033) increased, systolic blood pressure, resting heart rate and percent body fat decreased, respectively. Sedentary behavior was also significantly correlated with neighborhood environment variables. The amount of time spent sitting in a motor vehicle each day increased as attractiveness for walking (r = .150, p = .006) and cycling (r = .142, p = .009) increased, yet the amount of time spent sitting on the weekend, excluding time spent sitting in a motor vehicle, decreased as attractiveness of cycling (r = −.108, p = .050) and safety for walking (r = −.110, p = .044) increased.

### Regression Models

A summary of significant associations between neighborhood factors and cardiometabolic risk factors is presented in Table 1. Linear regression models indicated a positive linear association between crossing aids and BMI (β = .124, t = 2.25, p = .025), suggesting that having more crossing aids in the neighborhood was associated with higher resident BMI. Having more pedestrian sidewalk buffers (β = −.101, t = −1.94, p = .053) was associated with lower systolic blood pressure, consistent with hypotheses. However, in contrast, having more crossing aids (β = .098, t = 1.94, p = .053) was associated with higher systolic blood pressure. Having more traffic control devices was associated with lower resting heart rate (β = −.135, t = −2.45, p = .015), consistent with hypotheses.

**Table 1:** Direction of significant associations between PEDS variables and cardiometabolic risk factors.

PEDS Variable	BMI	Systolic blood pressure	Resting heart rate	Time spent sitting in motor vehicle
Crossing Aids	+	+		
Sidewalk Buffers		−h		
Traffic Control Devices			−h	
Attractive for Walking				+^h^
Attractive for Cycling				+^h^
Safe for Walking				+^h^
Safe for Cycling				+^h^

+ = positive association,

− = negative association,

h consistent with hypotheses/expected direction.

Consistent with hypotheses, attractiveness for walking (β = .154, t = 2.71, p = .007) and cycling (β = .148, t = 2.61, p = .010) and safety for walking (β = .115, t = 2.01, p = .045) and cycling (β = .122, t = 2.12, p = .035) were all associated with daily time spent sitting in a motor vehicle. As attractiveness and safety of the walking and cycling environment improved, time spent sitting in a motor vehicle, such as a car, train or bus, increased. Regression analyses are summarized in Table 2.

**Table 2:** Multiple Regression Models Predicting Cardiometabolic Risk Factors.

Cardiometabolic Risk Factor		Crossing Aids	Sidewalk Buffers	Traffic Control Devices	Attractive for walking	Attractive for cycling	Safe for walking	Safe for cycling
BMI								
	R^2^	.033						
	Unstandardized B	3.99						
	Standardized β	.124						
	t	2.25						
	p	.025						
Systolic blood pressure								
	R^2^	.183	.183					
	Unstandardized B	5.08	−2.73					
	Standardized β	.098	−.101					
	t	1.94	−1.94					
	p	.053	.053					
Resting heart rate								
	R^2^			.050				
	Unstandardized B			−5.60				
	Standardized β			−.135				
	t			2.54				
	p			.015				
Time spent sitting in a motor vehicle								
	R^2^				.038	.036	.028	.029
	Unstandardized B				.295	.281	.216	.220
	Standardized β				.154	0.148	.115	.122
	t				2.08	2.61	2.01	2.12
	p				.007	0.01	.045	.035

## Conclusions

This study investigated relationships between micro-environmental, street scale elements and cardiometabolic risk factors of sedentary behavior, BMI, percent body fat, blood pressure and resting heart rate among African American and Hispanic or Latina women. Neighborhood micro-environmental elements that were proximal to the residence had an important and complex influence on cardiometabolic risk factors. Consistent with hypotheses, living in neighborhoods with more pedestrian sidewalk buffers and traffic control devices was associated with improved blood pressure and resting heart rate, potentially indicative of a healthier lifestyle (e.g., more physical activity, less stress). Sidewalk buffers separate the road where vehicular traffic is flowing from the sidewalk or path where pedestrians are. Traffic control devices slow and regulate vehicular traffic, improving traffic predictability for other users of the street (e.g., pedestrians, cyclists). Both sidewalk buffers and traffic control devices have been significantly associated with improved sidewalk connectivity and pedestrian amenities [35], factors that likely improve the pedestrian experience–making it more enjoyable, more likely to be sustained, and less stressful. Improved pedestrian experiences then may lead to improved levels of cardiorespiratory fitness, even when BMI is above normal. [36] It is also possible that neighborhoods with these features are more tranquil, contributing indirectly to cardiometabolic outcomes through as yet unspecified biologic pathways. For example, quieter neighborhoods may produce improved sleep quality which may in turn lead to lower blood pressure and resting heart rate [37].

In contrast, neighborhoods with more crossing aids were neighborhoods where women with higher BMIs were likely to live. This might be explained by previous work that has shown that while pedestrian crossing aids are associated with improved sidewalk connectivity and pedestrian amenities, they are also associated with higher traffic volume and speed limit [35] producing an unfavorable and stressful environment for outdoor physical activity. Van Dyck and colleagues (2010) also found that residents of high-walkable neighborhoods (more dense and ‘urban’) reported more sitting time than those of low-walkable neighborhoods. [16] Thus, perhaps women in these neighborhoods were simply not using neighborhood streets to do physical activity which is typically associated with lower BMI. Crawford and colleagues found perceived traffic volume to be inversely related to physical activity in children, [38] which may suggest a similar inverse association between traffic control devices, such as stop signs and street lights, and BMI. Other work has shown that proximity to safe and attractive destinations for physical activity were not consistently associated with BMI [39] and blood pressure. [40] Taken together these findings suggest more work needs to be done to disentangle the complex relationships among street scale elements and cardiometabolic factors.

Our models showed that women who lived in neighborhoods that were most well suited for physical activity in terms of safety and attractiveness were the same women who spent the longest period of time in their car. It may be that the safer and more attractive neighborhoods were in suburban areas, which are often more aesthetic, less utilitarian and located farther away from commercial and employment hubs. In a large and sprawling city like Houston, Texas, with multiple central business hubs, it can be difficult to tease out whether a neighborhood is “suburban” based on traditional indicators such as distance from city center or population density. However, consistent with our finding that women were more likely to report time sitting in a motor vehicle if they lived in safer and more attractive neighborhoods, population density was positively correlated with path connectivity and number of travel lanes (which may indicate a more utilitarian or “urban” neighborhood), and negatively correlated with time spent sitting in a motor vehicle on weekdays, and total time spent spend sitting in a motor vehicle (analyses not shown). Thus it may be that the neighborhoods which were safer and more attractive for walking and cycling may have been less dense or ‘suburban’ requiring a longer commute time and leading to more time sitting in a motor vehicle. Car ownership and greater time spent in the car has been associated with sedentary lifestyle and increased obesity; [41], [42] thus, interventions are needed that address commuting and the neighborhoods in which commuters reside.

Although not shown in regression summaries, it is important to note that in predicting BMI, in nearly all regressions, educational attainment was significantly associated with BMI, washing out potential bivariable relationships between neighborhood street scale elements and BMI. This is noteworthy; although age and ethnicity are fixed factors, educational attainment can be modified, potentially improving numerous cardiometabolic outcomes in minority women. Educational attainment brings greater knowledge of health and healthy habits, as well as greater potential for acquiring wealth, access to improved goods and services, and may reduce day to day stressors that contribute to cardiometabolic outcomes. [43] Improving educational access and attainment for women should be an important goal not only in and of itself, but also for improving health outcomes.

Strengths of the current study include systematic protocols and detailed in-person audits of a large sample of neighborhoods and street scale elements. This study also followed a sizeable sample of African American and Hispanic or Latina women residing in neighborhoods widely distributed throughout the cities of Houston and Austin, Texas, maximizing geographic variability and increasing generalizability, and is among the first to study these kinds of factors in relationship to cardiometabolic risk factors in these samples. However, since our participant (and as a result, our neighborhood) sample consisted of volunteers, it was impossible to eliminate all selection bias.

This study relied on cross-sectional data, which cannot determine causation or the direction of associations. For example, due to the presence of selection bias it cannot be determined whether neighborhood with more pedestrian buffers and traffic control devices led to lower systolic blood pressure and resting heart rate, or if people with lower systolic blood pressure and resting heart rate choose to live in neighborhoods that possess these elements. As this was a secondary data analyses, we had to rely on existing measures of sedentary behavior in the data set to answer our research questions, and future studies should consider additional measures of sedentary behavior. Our findings are generalizable only to overweight, minority women. Although our findings are generalizable to only urban and suburban neighborhoods, the quality of the data and consistency of the findings may inform research and practice among other populations and neighborhoods. The linkage between street scale elements and cardiometabolic risk factors is multifaceted and should be addressed in future interventions and programming. Additional measures of attractiveness and safety to identify specific aesthetic and safety features related to BMI, percent body fat, blood pressure, resting heart rate and sedentary behavior are needed, and longitudinal studies are a critical next step to determine how features of the built environment may influence sedentary behavior and other cardiometabolic risk factors over time. This study showed that residence in areas that are safer and more attractive for walking and bicycling is associated with more time spent sitting in motor vehicles, but living in safe and attractive places may also provide some health benefits such as lower blood pressure and resting heart rate. Future interventions are needed to address contemporary lifestyles that may necessitate a “commuting culture.”
